# Previous radiotherapy improves treatment responses and causes a trend toward longer time to progression among patients with immune checkpoint inhibitor-related adverse events

**DOI:** 10.1007/s00262-023-03494-4

**Published:** 2023-07-24

**Authors:** Anna Jokimäki, Henna Hietala, Jasmiini Lemma, Hanna Karhapää, Anna Rintala, Jari-Pekka Kaikkonen, Kaisa Sunela, Eva Boman, Arja Jukkola, Satu Tiainen, Jan Seppälä, Aino Rönkä, Heikki Hakkarainen, Aarno Kärnä, Sanna Iivanainen, Jussi Koivunen, Päivi Auvinen, Micaela Hernberg, Milla Kuusisto, Tuomas Selander, Outi Kuittinen

**Affiliations:** 1 Department of Oncology and Radiotherapy, Oulu University Hospital, Oulu, Finland; 2 Faculty of Health Sciences, Institute of Clinical Medicine, University of Eastern Finland, Kuopio, Finland; 3 Medical Research Center Oulu, Oulu University Hospital, Oulu, Finland; 4 Department of Oncology, Comprehensive Cancer Center, Helsinki University Hospital, Helsinki, Finland; 5 University of Helsinki, Helsinki, Finland; 6 Faculty of Medicine and Health Technology, Tampere Cancer Center, Tampere University, Tampere, Finland; 7 Department of Oncology, Tampere University Hospital, Tampere, Finland; 8 Center of Oncology, Kuopio University Hospital, Kuopio, Finland; 9 Department of Oncology, Hospital of Central Finland Nova, Jyvaskyla, Finland; 10 Department of Hematology, Oulu University Hospital, Oulu, Finland; 11 Science Service Center, Kuopio University Hospital, Kuopio, Finland

**Keywords:** Immune checkpoint inhibitors, Immune-related adverse events, Radiotherapy, Overall response rate

## Abstract

**Background:**

Immune-related adverse events (irAEs) are frequently encountered by patients during immune checkpoint inhibitor (ICI) treatment and are associated with better treatment outcomes. The sequencing of radiotherapy (RT) and ICIs is widely used in current clinical practice, but its effect on survival has remained unclear.

**Methods:**

In a real-world multicenter study including 521 patients who received ICI treatment for metastatic or locally advanced cancer, RT schedules and timing, irAEs, time to progression, overall survival, and treatment responses were retrospectively reviewed.

**Results:**

Patients who received previous RT and developed irAE (RT +/AE +) had the best overall response rate (ORR 44.0%). The ORR was 40.1% in the RT −/AE + group, 26.7% in the RT −/AE − group and 18.3% in the RT + /AE − group (*p *< 0.001). There was a significantly longer time to progression (TTP) in the RT + /AE + group compared to the RT −/AE − and RT + /AE − groups (log rank *p *= 0.001 and *p *< 0.001, respectively), but the trend toward longer TTP in the RT + /AE + group did not reach statistical significance in pairwise comparison to that in the RT −/AE + group. Preceding RT timing and intent had no statistically significant effect on TTP. In a multivariate model, ECOG = 0 and occurrence of irAEs remained independent positive prognostic factors for TTP (HR 0.737; 95% CI 0.582–0.935; *p *= 0.012, and HR 0.620; 95% CI 0.499–0.769; *p *< 0.001, respectively).

**Conclusions:**

Better ORR and a trend toward longer TTP were demonstrated for patients with RT preceding ICI treatment and development of irAEs, which suggests that RT may boost the therapeutic effect of immunotherapy in patients with metastatic cancers.

**Supplementary Information:**

The online version contains supplementary material available at 10.1007/s00262-023-03494-4.

## Background

Immune checkpoint inhibitors (ICIs) have become the backbone of treatment for several metastatic cancers. The most common indications are metastatic or locally advanced non-small-cell lung cancer (NSCLC), metastatic melanoma, renal cell carcinoma (RCC) and urothelial cancer. The mechanism of action of ICIs includes binding of a monoclonal antibody to either PD-1 or CTLA-4 on the surface of T cells or to PD-L1 on tumor cells, which prevents their inhibitory action on T cells and enhances the immune response against cancer [1]. ICI treatment may lead to long-term survival of cancer patients, with the 5-year overall survival in metastatic diseases ranging from 16% (NSCLC) to 34% (cutaneous melanoma) [2]. Grade ≥ 3 immune-related adverse events (irAEs) occur in up to 10–15% of patients receiving anti-PD1 monotherapy and over half of patients receiving combination therapy (nivolumab-ipilimumab), and they can limit the possibility of continuing ICI treatment [3, 4];.

Radiotherapy (RT), with either curative or palliative intent, has been used for decades in cancer treatment. Recently, multimodal treatment design, with RT courses preceding and overlapping with ICI treatment, has emerged in modern oncology practice despite the fact that survival and safety data for these combinations are still scarce. The first phase III study to support a beneficial effect of RT and ICI sequencing was the PACIFIC study, in which administering durvalumab after chemoradiation in stage III NSCLC improved progression-free survival and overall survival (PFS and OS, respectively) [5, 6]. However, avelumab did not demonstrate enhanced survival outcomes after chemoradiation for locally advanced head/neck squamous cell carcinoma [7]. For multiple phase III studies combining (chemo)radiotherapy with ICIs, such as KEYNOTE-975 in esophageal carcinoma [8], the results are still pending. Concurrent or sequential administration of RT and pembrolizumab augmented PFS and OS in a pooled analysis of metastatic NSCLC studies [9]. Real-world evidence also supports the idea that the use of stereotactic radiation and immunotherapy in melanoma brain metastasis patients may enhance survival [10].

Purpose of this study was to retrospectively investigate the effect of previous RT and immune-related adverse events (irAEs) on treatment responses and survival outcomes in a real-world population of ICI-treated patients with metastatic or locally advanced malignancies.

## Materials and methods

### Study population

The study population included 590 cancer patients treated with ICIs at five Finnish oncology centers: the Helsinki, Kuopio, Oulu and Tampere University Hospitals and the Hospital of Central Finland Nova. Patients started their therapy between January 2014 and May 2019. The study was approved by the Ethics Committee of Northern Osthrobothnia Hospital District. Data were collected from the hospital records and RT treatment-planning systems, and included patient and disease baseline characteristics, ICI infusion and timing records, disease outcomes, and RT target, dose and timing records.

**Fig. 1:**
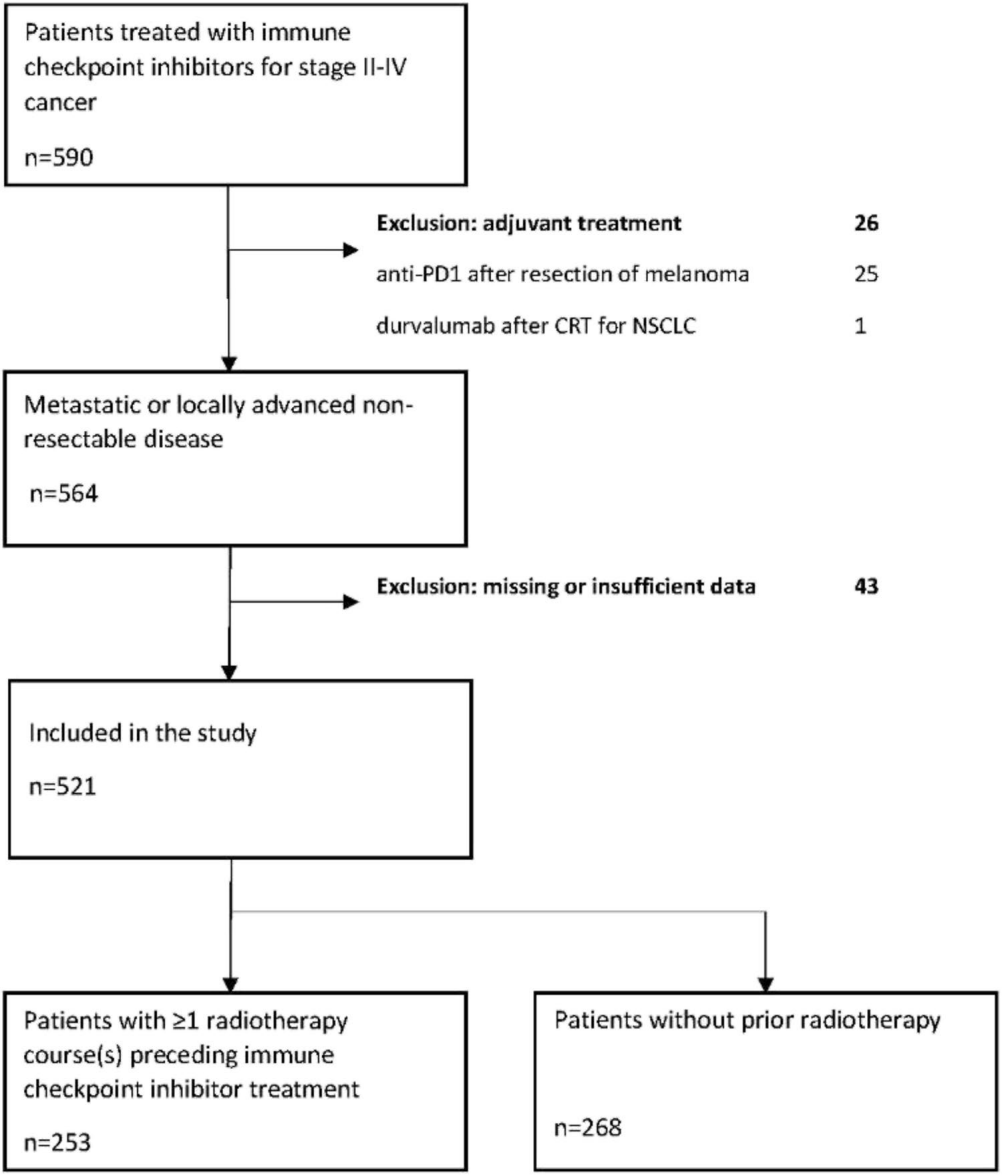
Flowchart of patient selection

Only patients with stage III-IV metastatic or locally advanced cancer at the time of ICI therapy were included. The only patient included with stage II disease had Hodgkin lymphoma (Ann Arbor stage II). We excluded 1 patient who received durvalumab treatment after radical chemoradiotherapy for stage III NSCLC and 25 patients treated with adjuvant nivolumab or pembrolizumab after resection of stage III cutaneous melanoma. Forty-three patients had to be excluded due to substantial lack of data, such as missing ICI and/or radiotherapy timing information. After these exclusions, *n *= 521 patients were accepted for further analyses (flowchart in Fig. 1).

### Radiotherapy data collection

The timing, schedule, total dose and fractionation were designated separately for each RT course. Altogether, 788 RT courses were administered in the whole population, and of those, 376 of the courses (47.7%) were given before the start of ICI treatment; the rest (412, 52.3%) were given concomitantly with ICI or after ICI treatment discontinuation. In the further survival analyses, only RT courses preceding ICI were included.

RT courses preceding ICI (*n *= 376) were classified (in co-operation with a medical radiation physicist, H. Hietala) as (1) given with palliative intent and variable fractionation (51.1%, *n *= 192 courses), (2) given with definitive/curative intent and conventional fractionation (31.1%, *n *= 117 courses), or (3) given as stereotactic intracranial and extracranial radiotherapy (8.2%, *n *= 31 courses). A total of 9.6% of the RT courses could not be classified due to missing data. For cases missing specific RT start or end dates, the dates were estimated, taking into consideration the fractionation and total dose of the RT course.

### Immune-related adverse event data

Data regarding irAEs during and after ICI treatment were collected from medical records by the collaborators at each hospital clinic. The irAEs were graded using the Common Terminology Criteria for Adverse Events** (**CTCAE) version 5.0 (National Cancer Institute, 2017).

### Statistics

All analyses were performed by IBM SPSS Statistics for Windows versions 25 or 27. Data were expressed as frequencies with percentages and comparisons were executed by chi-square or Fisher’s exact tests. Survival analyses were performed using the Kaplan–Meier method with the log-rank test. Multivariate analysis was performed using Cox regression and hazard ratios (HR) with 95% confidence intervals were reported. Two different Cox regression models were applied, the first one with only irAE and RT, and the second one including also other prognostic factors. Pairwise analysis was used to compare differences between treatment groups. *P* values < 0.05 were considered statistically significant.

Overall survival (OS) was calculated from the date of ICI treatment initiation to the date of death for any reason or the last follow-up date. Time to progression (TTP) was calculated from the date of ICI treatment initiation to the date of disease progression or disease-related death or the last follow-up date, whichever occurred first. If the patient received repeat ICI treatment or was treated with another ICI or combination therapy later on during follow-up, the follow-up ended at the start of the repeat or second-line ICI treatment.

## Results

### Overall patient and treatment characteristics

The baseline characteristics of all patients stratified according to receipt of previous radiotherapy (given/not given) are presented in Table 1. The median age of the patients was 65 years (range 20–85 years). There was a male predominance in this population, *n *= 331 (63.4%). The majority of the patients (80.1%) had good performance status [ECOG (Eastern Cooperative Oncology Group) performance status 0–1]. The most common treatment indications were metastatic cutaneous melanoma, metastatic/locally advanced NSCLC, and metastatic renal cell carcinoma. Other cancers were merged into one group (*n *= 82, 15.7%) due to the small numbers of the designated cancer types, including urothelial carcinoma, Hodgkin lymphoma, head/neck squamous cell carcinoma, gastric carcinoma, breast cancer, prostate cancer, Merkel cell carcinoma, uveal melanoma, mucosal melanoma, anal squamous cell carcinoma, lymphoblastic lymphoma, follicular lymphoma, cervical carcinoma, and alveolar soft tissue sarcoma.

**Table 1:** The characteristics of patients divided by previously given radiotherapy

	All patients (*n *= 521)	Patients without prior radiotherapy (*n *= 268)	Patients with prior radiotherapy (*n *= 253)	*p* (Chi-square)
*Age*				
< 65 years	278 (53.4%)	153 (57.1%)	125 (49.4%)	0.048^e^*
≥ 65 years	243 (46.6%)	115 (42.9%)	128 (50.6%)	
*Gender*				
Female	190 (36.5%)	94 (35.1%)	96 (37.9%)	0.278^e^*
Male	331 (63.5%)	174 (64.9%)	157 (62.1%)	
*ECOG*				
0	187 (35.9%)	108 (40.3%)	79 (31.2%)	0.013*
1	230 (44.1%)	110 (41.0%)	120 (47.4%)	
2	36 (6.9%)	11 (4.1%)	25 (9.9%)	
3	1 (0.2%)	0 (0.0%)	1 (0.4%)	
Not available	67 (12.9%)	39 (14.6%)	28 (11.1%)	
*Cancer diagnosis*				
Cutaneous melanoma	194 (37.2%)	114 (42.5%)	80 (31.6%)	< 0.001*
Non-small-cell lung cancer	144 (27.6%)	66 (24.6%)	78 (30.8%)	
Renal cell carcinoma	101 (19.4%)	56 (20.9%)	45 (17.8%)	
Urothelial carcinoma	20 (3.8%)	14 (5.2%)	6 (2.4%)	
Hodgkin lymphoma	16 (3.1%)	8 (3.0%)	8 (3.2%)	
Head/neck squamous cell Carcinoma	15 (2.9%)	0 (0.0%)	15 (5.9%)	
Other cancers^a^	29 (5.6%)	10 (3.6%)	19 (7.6%)	
Cancer type unknown	2 (0.4%)	0 (0.0%)	2 (0.8%)	
*Stage*				
II	1(0.2%)	0 (0.0%)	1 (0.4%)	0.057
III	16 (3.1%)	4 (1.5%)	12 (4.7%)	
IV	504 (96.7%)	264 (98.5%)	240 (94.9%)	
*Immune checkpoint inhibitor*				
Nivolumab	303 (58.2%)	160 (59.7%)	143 (56.5%)	0.711
Pembrolizumab	176 (33.8%)	88 (32.8%)	88 (34.8%)	
Atezolizumab	25 (4.8%)	11 (4.1%)	14 (5.5%)	
Other immune checkpoint inhibitor^b^	16 (3.1%)	9 (3.4%)	7 (2.8%)	
Treatment not specified	1 (0.2%)	0 (0.0%)	1 (0.4%)	
*Line of therapy*^*c*^				
First line	172 (33.5%)	108 (40.6%)	64 (25.9%)	0.001*
Second line	196 (38.2%)	87 (32.7%)	109 (44.1%)	
Third or later line	145 (28.3%)	71 (26.7%)	74 (30.0%)	
*Treatment response*				
Progressive disease	246 (47.2%)	116 (43.3%)	130 (51.4%)	0.102
Stable disease	87 (16.7%)	52 (19.4%)	35 (13.8%)	
Partial response	109 (20.9%)	60 (22.4%)	49 (19.4%)	
Complete response	57 (10.9%)	30 (11.2%)	27 (10.7%)	
No data available	22 (4.3%)	10 (3.7%)	12 (4.8%)	
*Immune checkpoint inhibitor-related adverse events*				
Skin adverse events	84 (16.1%)	48 (17.9%)	36 (14.2%)	0.153
Thyroid-related adverse events	67 (12.9%)	36 (13.4%)	31 (12.3%)	0.394
Pneumonitis	39 (7.5%)	16 (6.0%)	23 (9.1%)	0.120
Colitis	35 (6.7%)	19 (7.1%)	16 (6.3%)	0.427
Hepatitis	31 (5.9%)	17 (6.3%)	14 (5.5%)	0.415
Arthritis/arthralgia	19 (3.6%)	13 (4.9%)	6 (2.4%)	0.099
Nephritis	13 (2.5%)	7 (2.6%)	6 (2.4%)	0.540
Myocarditis	10 (1.9%)	3 (1.1%)	7 (2.8%)	0.148
Other irAEs^d^	64 (12.3%)	39 (14.6%)	25 (9.8%)	0.066

The statistically significant *p* values are marked with an asterisk

^a^“Other cancers” refers to a heterogeneous group of cancers, including gastric, breast and prostate cancers, Merkel cell carcinoma, uveal melanoma, mucosal melanoma, anal squamous cell carcinoma, lymphoblastic lymphoma, follicular lymphoma, cervical carcinoma and alveolar soft tissue sarcoma

^b^“Other immune checkpoint inhibitor treatment” refers to ipilimumab, nivolumab + ipilimumab, durvalumab, avelumab, tremelimumab, or nivolumab + investigatory ICI combination

^c^For eight patients, the treatment line was not specified

^d^“Other irAEs” = other immune-related adverse events refers to having at least one rare event, including (n in the whole population): myositis/myalgia (*n *= 15), diabetes (*n *= 10), hypocortisolism (*n *= 9), infusion reactions (*n *= 7), pancreatitis (*n *= 7), hypophysitis (*n *= 5), polyneuropathy (*n *= 4), fever/flu-like symptoms (*n *= 4), cholangitis (*n *= 2), iritis (*n *= 2), mucosal adverse events (*n *= 2), anemia (*n *= 1), and dizziness (*n *= 1)

^e^Fisher’s exact test *p* value

The most frequently used ICIs were nivolumab, pembrolizumab and atezolizumab. A total of 38.3% of patients received ICIs in the second line, 33.5% of patients received ICIs in the first line, and 28.2% of patients received ICIs in later lines of therapy. The median follow-up time was 14 months (range 0–62 months). The treatment response data (evaluated by collaborative physicians according to RECIST, Response Evaluation Criteria in Solid Tumors [11]) were complete for 95.8% (*n *= 500) of patients. Of these patients, 47.1% (*n *= 246) presented with progressive disease during ICI treatment. The overall response rate in the whole population was 31.8%, and 10.9% (*n *= 57) achieved complete response to ICI treatment. Twenty-six patients (5.0%) received at least one repeat/later-line ICI treatment.

### Comparison of patients who did or did not receive radiotherapy before immune checkpoint inhibitor treatment

Slightly older patients (Fisher’s exact *p *= 0.048) were given pre-ICI RT (Table 1). ECOG > 0 patients had received RT before ICI treatment more commonly: 57.7% of the patients in the pre-ICI RT group had ECOG performance status > 0 versus 45.1% in the no pre-ICI RT group (chi-square *p *= 0.013). Pre-ICI RT was more frequent in the NSCLC, head/neck squamous cell carcinoma and other cancer groups (chi-square *p *< 0.001). Additionally, patients with ≥ 1 prior systemic cancer treatment lines were more commonly treated with pre-ICI RT (chi-square *p *= 0.001). There was no statistically significant difference in the type of irAEs occurring in the patients who did and did not receive pre-ICI RT (*p* values presented in Table 1).

### Characteristics of the immune-related adverse events

The median time to the first irAE was 1.0 months (range 0–13 months). Almost half of the patients (48.6%, *n *= 253) had at least one irAE. Of those, 62.5% (*n *= 157) had only grade I-II irAEs, and 37.5% (*n *= 94) had one or more grade III-V irAEs, of which *n *= 3 were grade V irAEs. Among patients with pre-ICI RT, 15.8% developed at least one grade III-V irAE, while patients without previous RT more commonly had grade III-V irAEs (20.2%). None of the patients with grade V irAEs had received pre-ICI radiotherapy at the same anatomical area where the fatal irAE occurred. The irAEs were assessed by the collaborative physicians to be undisputedly connected with ICI treatment in 52.0% (*n *= 195) of cases and possibly connected with ICI treatment in 44.8% (*n *= 168) of cases, and the connection was not assessed or unclear in 3.2% (*n *= 12) of cases.

**Fig. 2:**
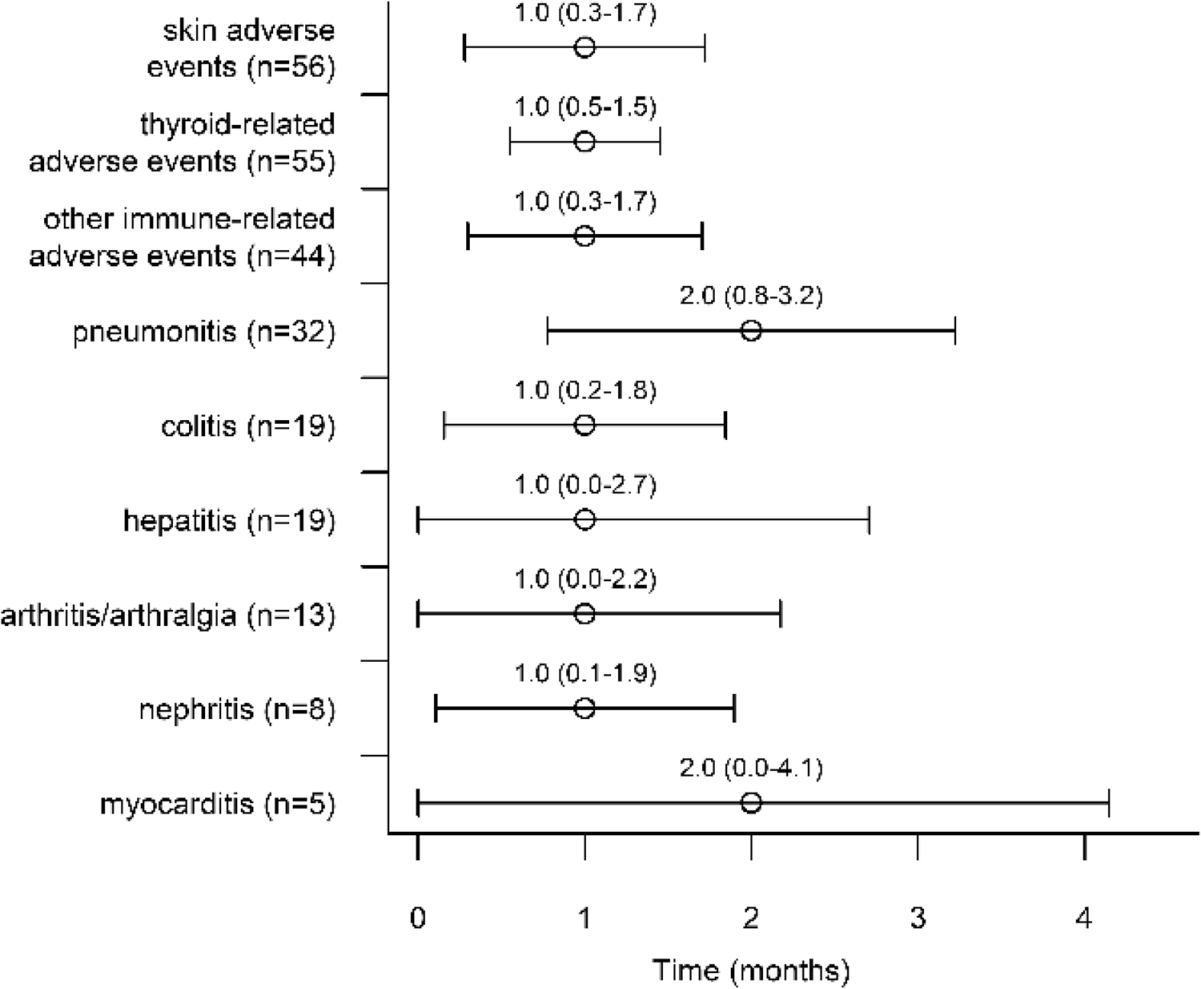
Timing of different immune-related adverse events

The timing and types of the first irAE are presented in Fig. 2. The timing did not significantly differ between different types of first irAEs (log rank *p *= 0.430). The most common first irAE was cutaneous irAE (*n *= 57, 22.5%), followed by thyroid-related irAE (*n *= 55, 21.7%) and pneumonitis (*n *= 32, 12.7%). A second irAE was reported in 17.0% of the patients (*n *= 88). The most common second irAE was also cutaneous irAE (*n *= 18, 20.5%), followed by colitis (*n *= 13, 14.8%) and thyroid-related irAE (*n *= 11, 12.5%). Three different irAEs were reported in 5.8% (*n *= 30) of the patients, and only 0.8% of patients had four irAEs. Cutaneous irAEs included eczematous, lichenoid, papulopustular, pemphigoid and psoriasiform cutaneous irAEs, pruritus, vitiligo, alopecia, urticaria, vasculitis, nail changes and systemic lupus erythematosus. Thyroid-related irAEs included subclinical and overt thyrotoxicosis/thyroiditis and/or subclinical or overt hypothyroidism.

### Overall association of radiotherapy and immune-related adverse events with disease outcomes

During ICI treatment, 67.4% patients (*n *= 351) and *n *= 64.8% (*n *= 164) patients who received pre-ICI RT experienced disease progression (including those with primary progression and patients with SD/PR/CR as the primary response to ICI). The median TTP on ICI treatment was 4.0 months (range 0–56 months).

In the following analyses, patients were stratified by receipt of RT preceding ICI (received/not received) and irAE status (at least one irAE/no irAEs). Table 2 presents the crosstabulation of best treatment responses (following RECIST criteria) among the RT and AE groups (chi-square *p *< 0.001). Overall, the patients with irAE had better response rates. The RT + /AE + group reached an overall response rate (ORR) of 44.0%, while the ORR of RT −/AE + group was 40.1%. In the RT −/AE − group, the ORR was 26.7%, while in the RT + /AE − group, the ORR was the poorest (18.3%). Complete responses (CR) were seen in 13.8% of patients in the RT + /AE + group and 13.1% of patients in the RT −/AE + group.

**Table 2:** Treatment response in patients who did or did not receive radiotherapy preceding immune checkpoint inhibitor treatment and who did or did not develop immune-related adverse events (chi-square *p *< 0.001)

Treatment response^a^	no RT, no AE	RT +, no AE	no RT, AE +	RT +, AE +
Total *n*	131	137	137	116
PD	67 (51.1%)	88 (64.2%)	49 (35.8%)	42 (36.2%)
SD	23 (17.6%)	17 (12.4%)	29 (21.2%)	18 (15.5%)
PR	23 (17.6%)	14 (10.2%)	37 (27.0%)	35 (30.2%)
CR	12 (9.2%)	11 (8.0%)	18 (13.1%)	16 (13.8%)
Not available	6 (4.6%)	7 (5.1%)	4 (2.9%)	5 (4.3%)

*PD* Progressive disease, *SD* Stable disease, *PR* Partial response, *CR* Complete response, *RT* Radiotherapy preceding immune checkpoint inhibitor treatment, *AE* Immune-related adverse event

^a^To evaluate treatment response, the RECIST criteria were used

**Fig. 3:**
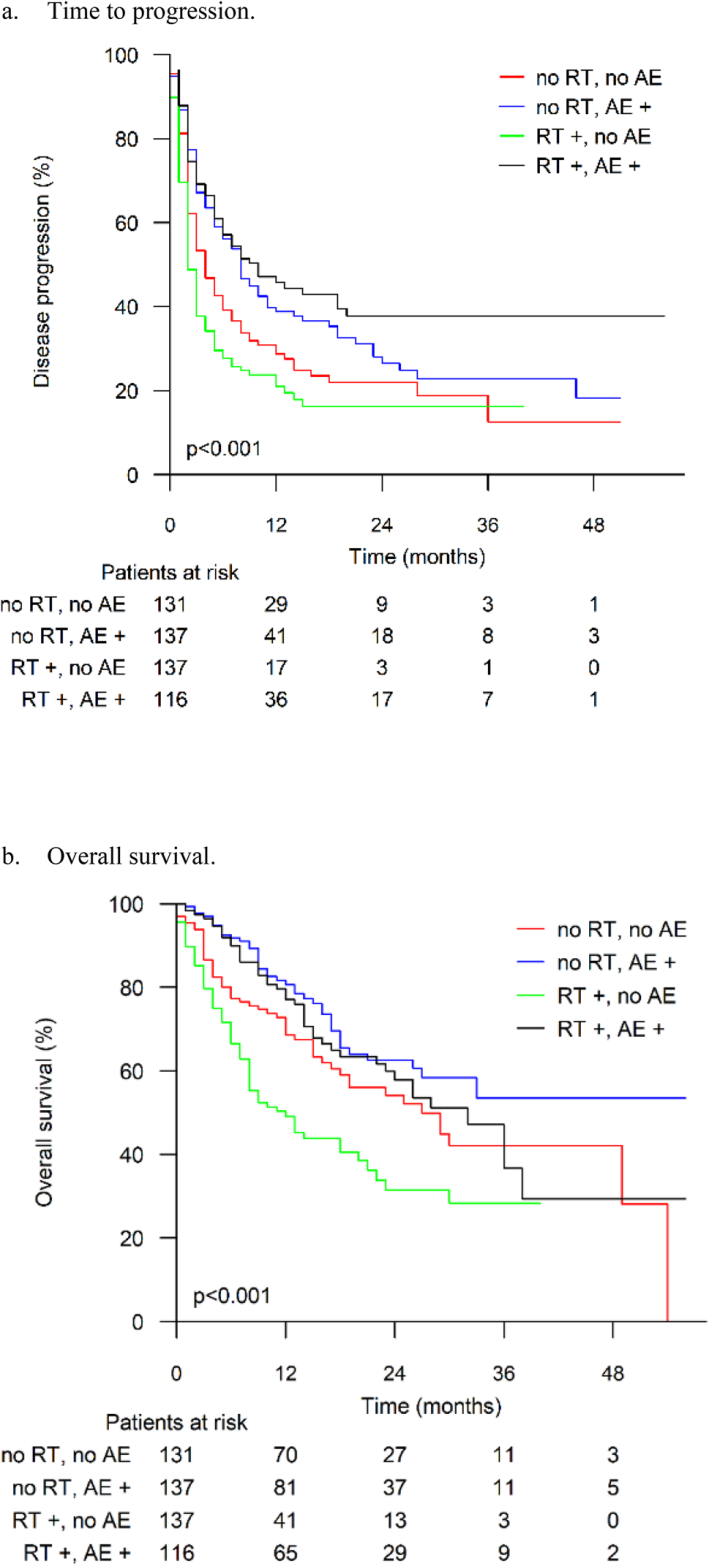
**a** Time to progression. **b** Overall survival results stratified by receipt of radiotherapy before immune checkpoint inhibitor treatment and development of immune-related adverse events. RT = radiotherapy preceding immune checkpoint inhibitor treatment, AE = immune-related adverse event

Figure 3a, b presents the TTP and OS analyses. In pairwise comparisons between groups, the RT + /AE + group had a significantly longer TTP than the RT −/AE − and RT + /AE − groups (log rank *p *= 0.001 and *p *< 0.001, respectively). However, there was no significant difference in TTP between the RT + /AE + and RT −/AE + groups (log rank *p *= 0.207), although the TTP curve seems to hint at a larger proportion of long-term survivors in the RT + /AE + group.

The combined effect of RT and irAE on TTP was further analyzed in a Cox regression model. Those with RT + and AE + showed an enhanced TTP, with a HR 0.484 (95% CI 0.352–0.666), compared to those with RT − and AE + (HR 0.727; 95% CI 0.545–0.970). The difference was statistically significant (*p *= 0.025) in this two-factor model.

In the analysis of OS, the RT + /AE − group had worse results than the other groups (log rank *p *= 0.002 for RT −/AE −, *p *< 0.001 for RT −/AE + and *p *< 0.001 for RT + /AE +).

In Supplementary Table 1, the TTP results by cancer type, stratified by RT and AE statuses, are presented. Both cutaneous melanoma and NSCLC patients had statistically significant differences in TTP (log rank *p *= 0.002 and *p *= 0.015, respectively), with longer TTP in the RT −/AE + and RT + /AE + groups. No statistically significant TTP differences between the RT/AE groups were seen in renal cell carcinoma or other cancers (log rank *p *= 0.142 and *p *= 0.570, respectively).The Kaplan–Meier TTP curves are presented in Supplementary Fig. 1A–D.

We did not find a statistically significant difference in TTP according to irAE severity (log rank *p *= 0.119, Supplementary Fig. 2a). After exclusion of patients with grade III-V irAEs from the TTP analysis of the RT − and AE − groups, the results were similar to former results (log rank *p *< 0.001) (Supplementary Fig. 2b).

### Analysis of radiotherapy intent and timing

**Fig. 4:**
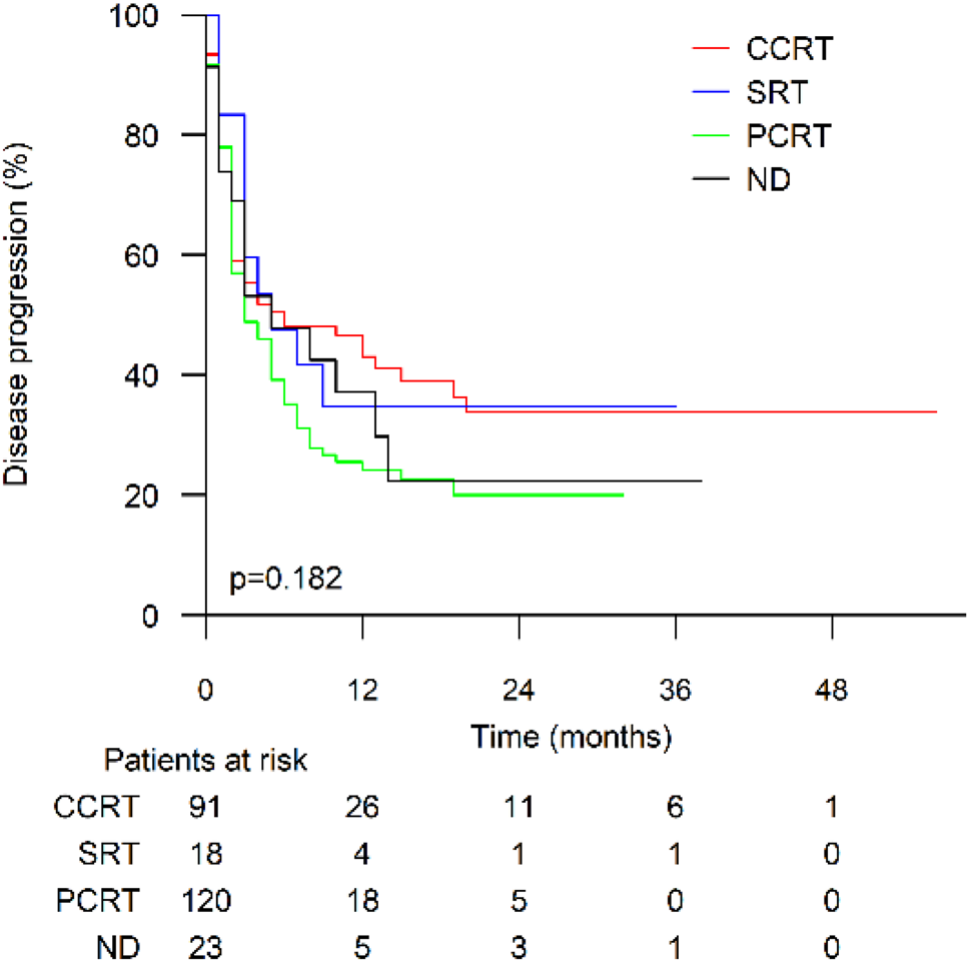
Time to progression in patients who received prior radiotherapy stratified by last radiotherapy intent. CCRT, curative intent and conventionally fractionated radiotherapy; SRT, stereotactic radiotherapy; PCRT, palliative intent and conventionally fractionated radiotherapy; ND, radiotherapy intent not defined

In the analysis of RT intent and timing, the data from the last RT course preceding ICI were used. The therapeutic intent of the last radiotherapy treatment before ICI (stratified as previously mentioned) did not have a significant effect on time to progression (log rank *p *= 0.182, Fig. 4).

**Fig. 5:**
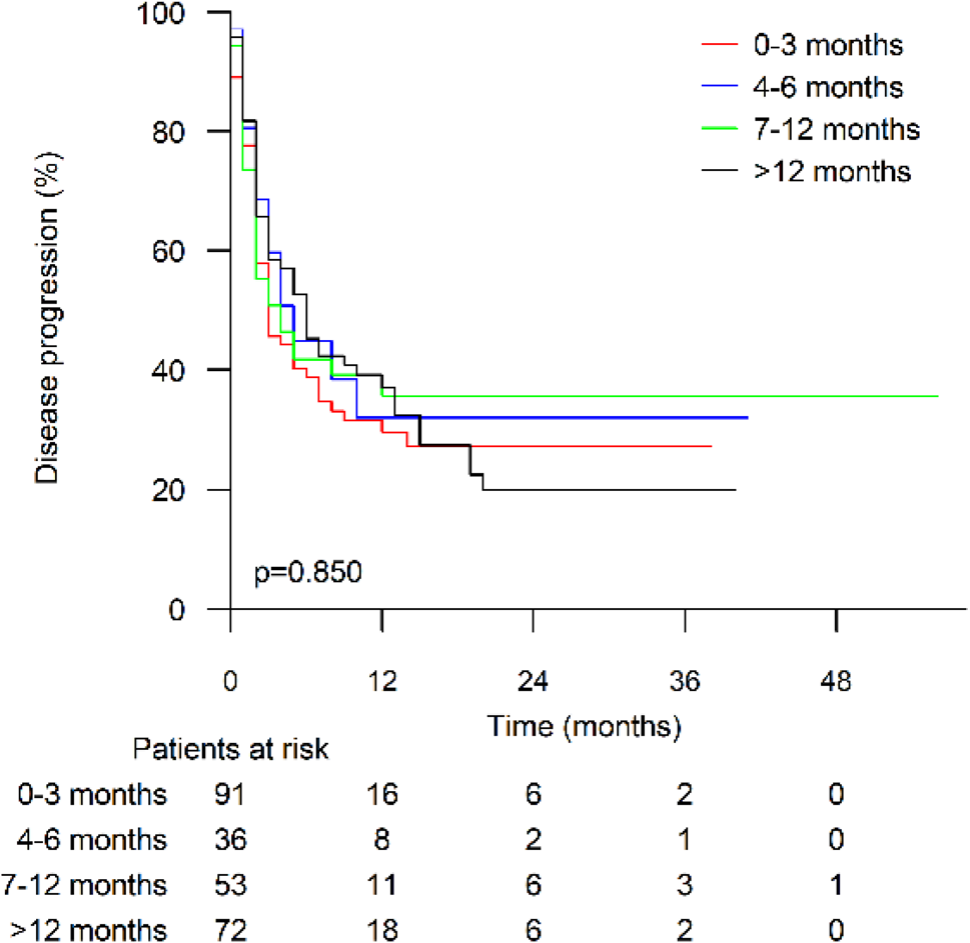
Time to progression stratified by time from last radiotherapy course to immune checkpoint inhibitor treatment start

RT was stratified as (1) 0–3 months, (2) 4–6 months, (3) 7–12 months or (4) > 12 months before the first ICI infusion. RT timing did not have any significant effect on TTP (log rank *p *= 0.850, Fig. 5).

A significant difference in TTP (log rank *p *< 0.001, Supplementary Fig. 3) was noted when RT was stratified as (1) not received, (2) received before ICI treatment only, (3) received concomitantly with ICI treatment only, (4) received both before and concomitantly with ICI treatment, (5) received after ICI treatment termination only, and (6) received both before and after ICI treatment. Patients receiving RT post-ICI had disease progression/oligoprogression during ICI treatment, accounting for their worse outcomes.

### Univariate analysis of the prognostic factors

In the univariate analysis of prognostic factors (Table 3), the occurrence of at least one irAE had a positive relationship with TTP (HR 0.594; 95% CI 0.480–0.735; *p *< 0.001). ECOG status 0 (HR 0.638; 95% CI 0.486–0.839; *p *= 0.001) and receipt of an ICI as a first-line therapy (HR 0.640; 95% CI 0.508–0.806; *p *< 0.001) also demonstrated a positive relationship with TTP. Age ≥ 65 years, sex, cancer stage IV (vs. II–III), RT preceding ICI (dichotomized as received/not received) and ICI therapy used (nivolumab/pembrolizumab/other immune checkpoint inhibitors) showed no statistically significant relationship with TTP (Table 3). Additionally, grade III–V irAEs (vs. no irAEs/grade I–II irAEs) had no significant effect (HR 0.866; 95% CI 0.658–1.140; *p *= 0.304). Cutaneous melanoma patients had a significantly longer TTP on ICI treatment than patients with other cancer types (HR 0.646; 95% CI 0.517–0.807; *p *< 0.001). In contrast, the TTP for patients with RCC was worse than that for other cancer types (HR 1.568; 95% CI 1.220–2.014; *p *< 0.001).

**Table 3:** Univariate and multivariate prognostic factor analysis results

Factor	Univariate analysis			Multivariate analysis		
	HR	95% CI	*P*^a^	HR	95% CI	*P*^a^
Age ≥ 65 years	1.138	0.922–1.403	0.229			
Female sex	0.090	0.661–1.031	0.098			
ECOG 0	0.638	0.486–0.839	0.001*	0.737	0.582–0.935	0.012*
*Cancer type*						
Melanoma	0.646	0.517–0.807	< 0.001*	0.887	0.671–1.201	0.421
NSCLC	1.076	0.850–1.363	0.543			
RCC	1.568	1.220–2.014	< 0.001*	1.230	0.930–1.627	0.146
Other	1.152	0.869–1.527	0.325			
Stage IV	2.029	0.960–4.289	0.064			
*ICI*						
Nivolumab	1.055	0.853–1.306	0.620			
Pembrolizumab	0.981	0.787–1.222	0.861			
Other	0.875	0.573–1.336	0.537			
First-line treatment	0.640	0.508–0.806	< 0.001*	0.788	0.591–1.051	0.106
Pre-ICI RT	1.066	0.864–1.315	0.550	1.021	0.283–1.265	0.851
Occurrence of irAE	0.594	0.480–0.735	< 0.001*	0.620	0.499–0.769	< 0.001*
≥ 1 grade III-V irAE	0.866	0.658–1.140	0.304			

The statistically significant *p* values are marked with an asterisk

*HR* Hazard ratio, *CI* Confidence interval, *ECOG* EASTERN Cooperative Oncology Group, *pre-ICI* Preceding immune checkpoint inhibitor treatment, *irAE* Immune-related adverse event

^a^*p* values from univariate and multivariate Cox regression analyses

### Multivariate analysis of the prognostic factors

In multivariate analysis (Table 3), ECOG = 0 and occurrence of at least one irAE remained independent positive prognostic factors (HR 0.737; 95% CI 0.582–0.935; *p *= 0.012, and HR 0.620; 95% CI 0.499–0.769; *p *< 0.001, respectively). Pre-ICI RT was also included in the multivariate analysis model but failed to show a statistically significant positive prognostic effect as a single variable (HR 1.021; 95% CI 0.283–1.265; *p *= 0.851).

## Discussion

In this large retrospective analysis, we analyzed the effect of receipt of previous RT and the occurrence of irAEs during ICI treatment on response rates and TTP in metastatic/locally advanced cancer. As previously reported [12], irAEs were associated with improved disease outcomes. RT seemed to strengthen this relationship, as patients who received previous radiotherapy and developed irAEs had a better response rate and a smaller hazard ratio for progression than those who developed irAEs but did not receive pre-ICI RT. In a multivariate Cox regression model, however, ECOG = 0 and occurrence of irAE remained the only significant prognostic factors.

ICIs have improved the treatment of locally advanced and metastatic solid tumors. There may even be a chance to cure a fraction of these patients with a disease that is conventionally considered to be fatal. However, for unknown reasons, only a proportion of patients respond to ICIs, and therefore, finding potential methods for improving response rates are necessary.

The occurrence of irAEs has been shown to have a positive effect on the survival outcomes of ICI-treated patients, and improvements in both PFS and OS were reported in a meta-analysis including patients with multiple treatment indications [12]. This survival benefit has been explained by the assumption that both normal tissue cells and tumor cells share the same antigens, leading to cross-reactivity. Radiotherapy, on the other hand, can lead to tumor-related antigen release, thus increasing the number of tumor-infiltrating lymphocytes and enhancing the T-cell mediated immune response [13]. RT can also activate dendritic cells [14] and enhance MHC1-mediated antigen presentation [15]. Through activation of these mechanisms, radiation can affect adjacent nonirradiated tumor cells, called the bystander effect, or even inhibit the growth of distant metastases by the abscopal effect.

Small phase I trials have reported promising results from the combination of RT and ICI treatment [16–18]. Moreover, in a subgroup analysis of 98 NSCLC patients in the KEYNOTE-001 trial [19], those who received previous radiotherapy demonstrated improved PFS and OS.

In this fairly large, multicenter retrospective study, we found that previous RT increased response rates and showed a trend toward improved TTP in ICI-treated patients. This was evident although more patients in the RT group had received several lines of therapy and had ECOG ≥ 1. In subgroup analysis, there was a significant difference in TTP only in melanoma and NSCLC, probably due to limited sample size.

Among patients who did not develop irAEs, those who received previous RT demonstrated worse outcomes in both TTP and OS, which is in line with their disadvantageous disease profile at the start of ICI treatment. In general, there were no differences in disease outcomes among patients who did or did not receive previous RT, but there was a difference in patients who developed irAEs and had received previous RT. This finding indicates that RT is probably itself not able to activate the patient’s immune system but instead enhances tumor immunology among immunologically responsive patients. Previous RT might be able to change a minority of immunologically cold tumors into hot ones. In this study, we did not derive any biological data regarding tumor immunology or circulating immune cells, and thus, no conclusions can be made regarding other factors interfering with treatment response.

The strengths of this study are the large sample size and real-life setting. Additionally, due to close collaboration with medical radiation physicists, we were able to gather detailed information on the RT given and analyze the timing and intent of RT. Unfortunately, the study also has the usual limitations of a retrospective design, including the absence of data on subsequent treatments after ICI discontinuation. Furthermore, despite the large sample size, in the subgroup analysis, we were unable to find significant minor differences related to, e.g., different radiotherapy intent. A longer follow-up period might have improved our ability to identify differences in TTP.

Several open questions remain regarding combining RT and ICI therapy. One important issue is the optimal timing of radiotherapy. In this study, the survival analyses were limited to the effect of former radiotherapies, and RT courses initiated after ICI treatment start were excluded from the analysis. We excluded these courses because in clinical practice, during/after discontinuation of ICI therapy, RT courses are usually given due to disease progression or oligoprogression, which is often associated with treatment discontinuation. Thus, inclusion of these courses may have biased the efficacy analysis. Additionally, there is evidence supporting the positive survival effects of RT preceding ICI treatment, such as the results from a trial of adjuvant durvalumab following chemoradiotherapy in stage III NSCLC [20]. The time from RT to the initiation of ICI treatment did not affect TTP, suggesting that the effect of radiotherapy may be long-lasting. Additionally, we did not find any impact of radiotherapy intent on TTP. However, despite a relatively large sample size, we were unable to identify minor effects in the subgroup analysis. Assuming the lack of association between radiotherapy and ICI timing can be verified in future studies, we hypothesize that the RT-induced improved tumor response is associated with the release of tumor antigens rather than changes in circulating immune cells because the levels of circulating cells are unlikely to be elevated longer periods after RT. In conclusion, this study provides an interesting point of view regarding associations between RT, irAEs, and ICI efficacy.

## Supplementary Information

Below is the link to the electronic supplementary material.

Supplementary file1 (PDF 420 KB)

## Data Availability

The datasets used and/or analyzed during the current study are available from the corresponding author on reasonable request.
