# Supplementary material for: Previous radiotherapy improves treatment responses and causes a trend toward longer time to progression among patients with immune checkpoint inhibitor-related adverse events
Source: Cancer Immunol Immunother. 2023 Jul 24;72(10):3337–47. doi: 10.1007/s00262-023-03494-4 (PMC10491510; doi:10.1007/s00262-023-03494-4)
Supplement: Supplementary file 1 — Supplementary file1 (PDF 420 KB) [file 262_2023_3494_MOESM1_ESM.pdf]

Supplementary Table 1. Median time to progression in different cancer types stratified by receipt of radiotherapy preceding immune checkpoint inhibitor treatment and occurrence of immune-related adverse events.

Cancer type	Median time to progression, months				p (log rank)
	no RT, no AE	RT+, no AE	no RT, AE+	RT+, AE+	
Cutaneous melanoma	6.0	3.0	14.0	20.0	0.001
Non-small-cell lung cancer	4.0	3.0	5.0	10.0	0.015
Renal cell carcinoma	3.0	2.0	3.0	5.0	0.142
Other cancers	6.0	2.0	8.0	5.0	0.570

RT, radiotherapy preceding immune checkpoint inhibitor treatment; AE, immune-related adverse event.

Supplementary Figure 1a-d. Time to progression by cancer type, stratified by receipt of radiotherapy preceding immune checkpoint inhibitor treatment and occurrence of immune-related adverse events.

a. Cutaneous melanoma.

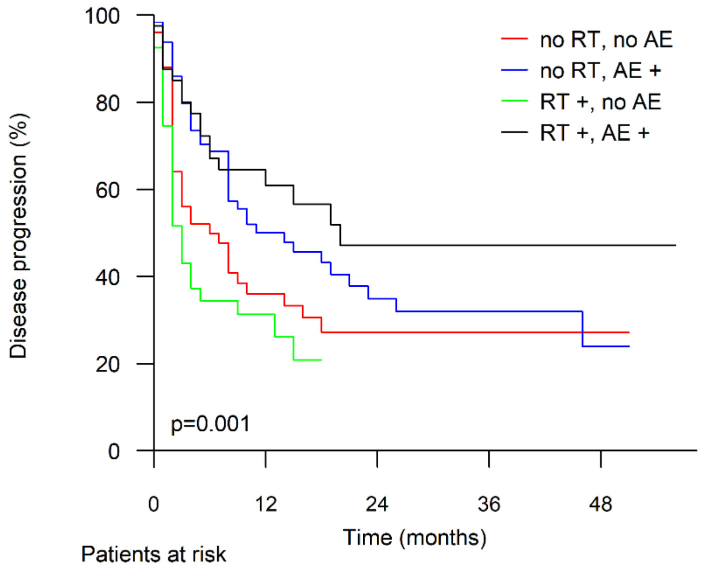

Patients at risk

no RT, no AE	50	15	6	2	1
no RT, AE +	64	24	12	7	3
RT +, no AE	40	6	0	0	0
RT +, AE +	40	18	8	5	1

RT, radiotherapy preceding immune checkpoint inhibitor treatment; AE= immune-related adverse event.

b. Non-small-cell lung cancer.

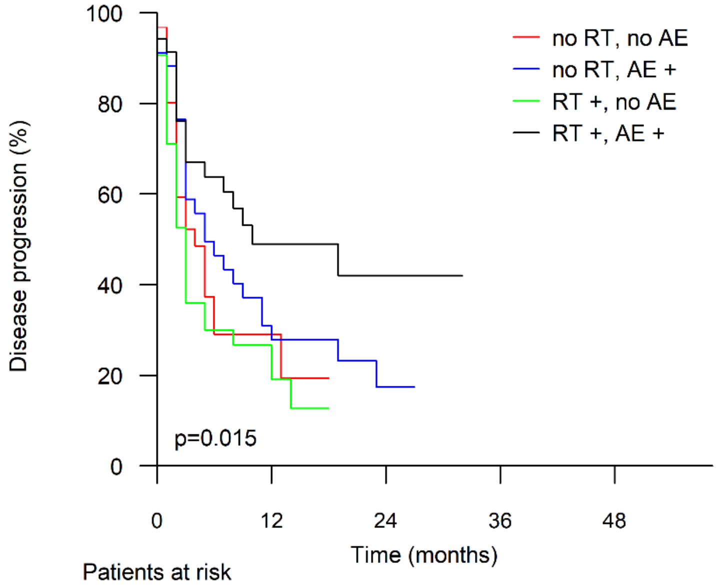

Patients at risk

no RT, no AE	32	5	0	0	0
no RT, AE +	34	10	3	0	0
RT +, no AE	43	7	0	0	0
RT +, AE +	35	10	5	0	0

c. Renal cell carcinoma.

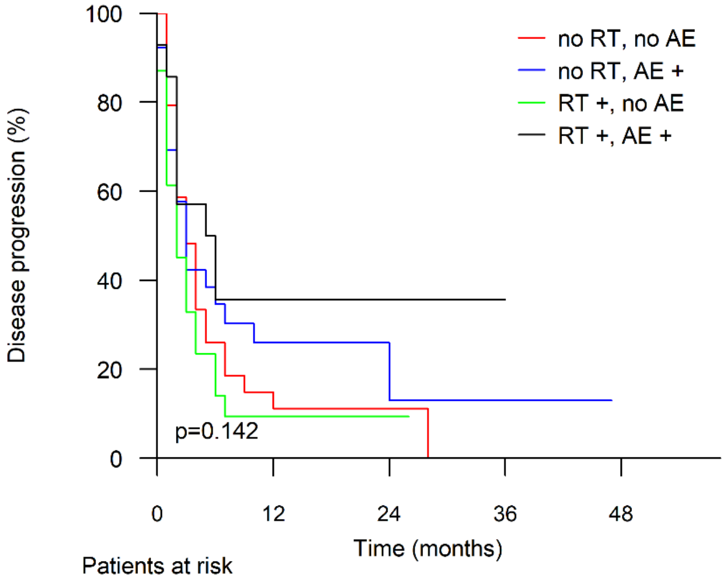

Patients at risk

no RT, no AE	30	4	1	0	0
no RT, AE +	26	6	2	1	0
RT +, no AE	31	2	1	0	0
RT +, AE +	14	3	2	1	0

d. Other cancers.

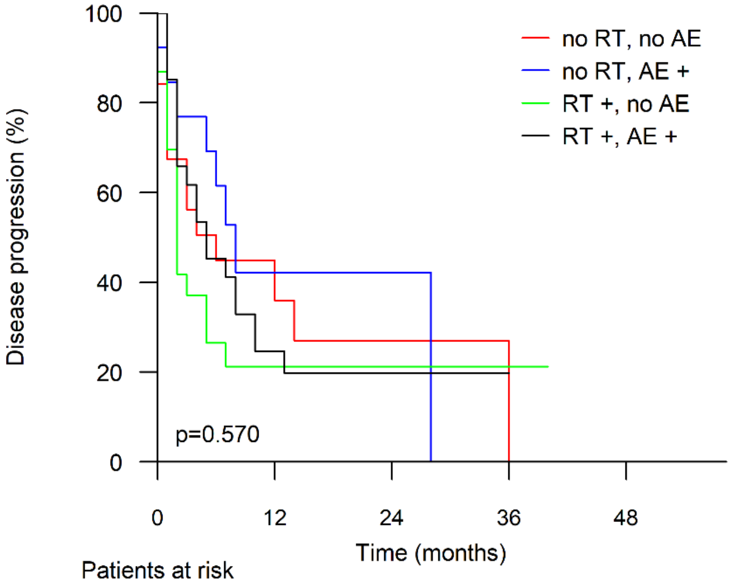

Patients at risk

	0	12	24	36	48
no RT, no AE	19	5	2	1	0
no RT, AE +	13	1	1	0	0
RT +, no AE	23	2	2	1	0
RT +, AE +	27	5	2	1	0

Supplementary Figure 2a. Effect of immune-related adverse event severity on time to progression (log rank p=0.119). 2b. Time to progression stratified by receipt of radiotherapy and occurrence of grade I-II immune-related adverse events (log rank p<0.001).

a.

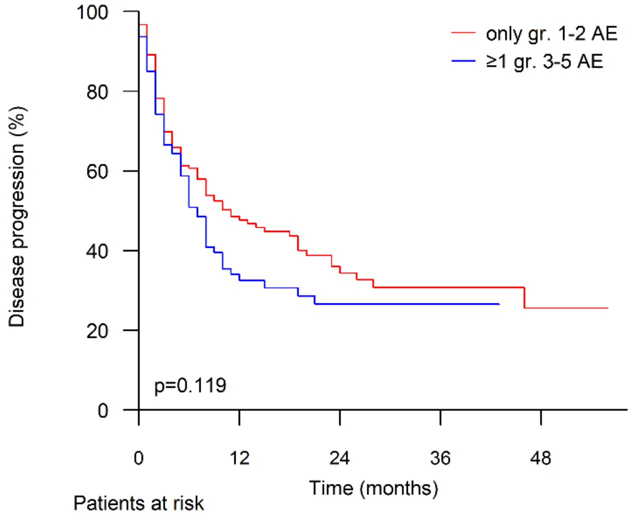

Patients at risk	0	12	24	36	48
only gr. 1-2 AE	157	54	22	9	4
≥1 gr. 3-5 AE	94	23	13	6	0

b.

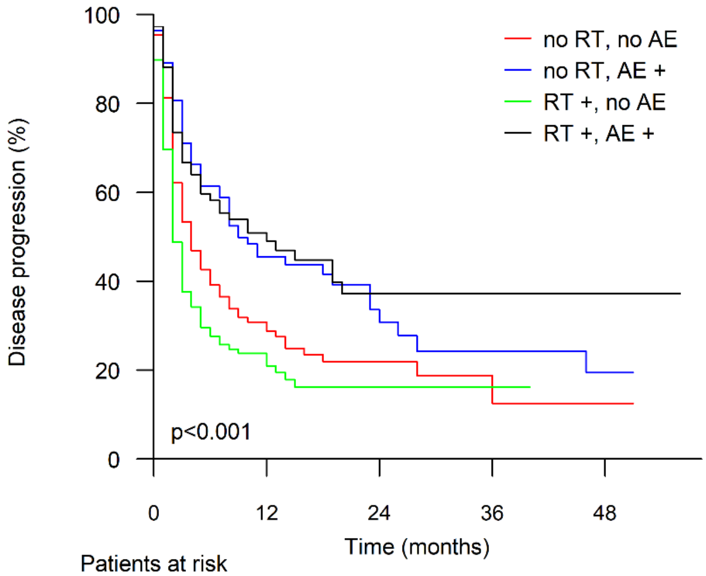

Patients at risk	0	12	24	36	48
no RT, no AE	131	29	9	3	1
no RT, AE +	83	27	12	5	3
RT +, no AE	137	17	3	1	0
RT +, AE +	76	27	10	4	1

Supplementary Figure 3. Time to progression stratified by radiotherapy timing (log rank p<0.001).

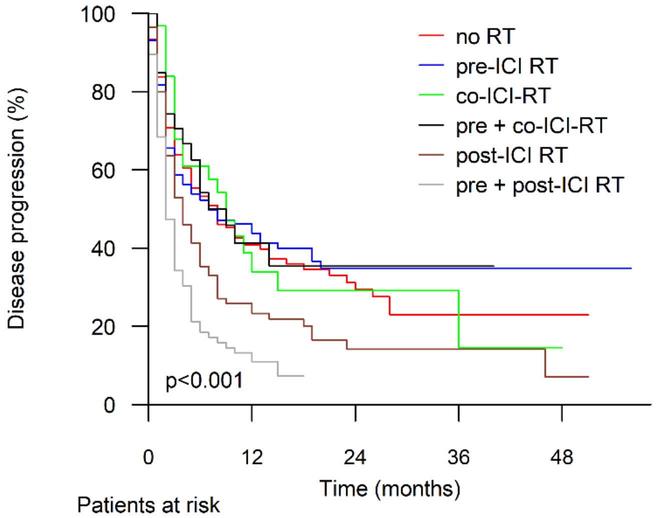

Patients at risk

	0	12	24	36	48
no RT	119	21	7	4	1
pre-ICI RT	119	41	15	7	3
co-ICI-RT	22	4	2	0	0
pre + co-ICI-RT	27	5	2	0	0
post-ICI RT	72	15	4	0	0
pre + post-ICI RT	68	14	4	2	1

RT, radiotherapy; pre-ICI, preceding immune checkpoint inhibitor treatment start; co-ICI, concomitantly with ICI; post-ICI, after ICI treatment discontinuation.
